# Coordinating Obstacle Avoidance of a Redundant Dual-Arm Nursing-Care Robot

**DOI:** 10.3390/bioengineering11060550

**Published:** 2024-05-29

**Authors:** Zhiqiang Yang, Hao Lu, Pengpeng Wang, Shijie Guo

**Affiliations:** 1 Academy for Engineering and Technology, Fudan University, Shanghai 200433, China; 2 College of Electronic Information and Automation, Tianjin University of Science and Technology, Tianjin 300222, China

**Keywords:** coordinating operation, obstacle avoidance, redundant dual-arm, nursing-care robots

## Abstract

Collision safety is an essential issue for dual-arm nursing-care robots. However, for coordinating operations, there is no suitable method to synchronously avoid collisions between two arms (self-collision) and collisions between an arm and the environment (environment-collision). Therefore, based on the self-motion characteristics of the dual-arm robot’s redundant arms, an improved motion controlling algorithm is proposed. This study introduces several key improvements to existing methods. Firstly, the volume of the robotic arms was modeled using a capsule-enveloping method to more accurately reflect their actual structure. Secondly, the gradient projection method was applied in the kinematic analysis to calculate the shortest distances between the left arm, right arm, and the environment, ensuring effective avoidance of the self-collision and environment-collision. Additionally, distance thresholds were introduced to evaluate collision risks, and a velocity weight was used to control the smooth coordinating arm motion. After that, experiments of coordinating obstacle avoidance showed that when the redundant dual-arm robot is holding an object, the coordinating operation was completed while avoiding self-collision and environment-collision. The collision-avoidance method could provide potential benefits for various scenarios, such as medical robots and rehabilitating robots.

## 1. Introduction

Nursing-care robots are efficient assistants for daily life actions such as home service and social guarantee [1,2]. Initially, single-arm robots were widely adopted for nursing-care robots due to their simple structure and low cost [3]. However, their limited operational flexibility and spatial efficiency make them underperform in complex or space-constrained environments [4]. To enhance the adaptability and flexibility of robots, researchers have introduced designs with redundant degrees of freedom (DoFs) [5,6], significantly enhancing the spatial reachability of single-arm robots and enabling them to be more flexible to complex environment. Despite these improvements, single-arm structures still exhibit performance bottlenecks when executing tasks that require dual-arm coordination, such as litter cleaning [7]. Consequently, further studies presented redundant dual-arm robots [8]. This design expanded the operational space [9], enhanced the capability to perform complex tasks through coordinated dual-arm operations [10], and improved the stability and efficiency of task execution [11]. Nevertheless, redundant dual-arm robots still face risks of self-collision and collisions with environmental obstacles (environment-collision).

Global path planning, as proposed by Kavraki et al. [12], is a collision-avoidance technology, which precomputes a path from the starting point to the endpoint to circumvent obstacles. This method is suitable for static environments where the positions of obstacles are completely known. In contrast, local path planning is more flexible, as shown by Hatib et al.’s research [13], which adapts the obstacle-avoidance path to accommodate dynamically changing environmental obstacles, and it is suitable for complex or frequently changing environments. Shen et al. [14] and Li et al. [15] used the local path planning method for redundant dual-arm robots to avoid environment-collision but without considering self-collision when performing coordinating operations. As an attempt, Wang et al. [16] and Wu et al. [17] considered the optimization of the robotic arm’s motion trajectories to avoid self-collision but overlooked the volumetric aspects of the arms. This oversight can lead to collisions between different parts of the robotic arm during actual operations. Additionally, according to Shi et al. [18] and Li et al. [19], recent obstacle avoidance strategies often cause the robotic arm to be unsmooth due to uneven joint trajectories in coordinated operations. Hence, for coordinated operations, there is still no suitable method to smoothly control motion while avoiding self-collision and environment-collision with the actual robot volume.

To address this issue, this study proposes an obstacle-avoidance algorithm for coordinated operations of redundant dual-arm robots based on their inherent kinematic properties. The main contribution of this work is as follows:

- A capsule-based algorithm was used to model the volume of the robotic arms, and a gradient projecting method was utilized to calculate the shortest distances (among the left arm, the right arm, and the environment), which achieved the actual body’s self-collision avoidance.
- By introducing a distance threshold to assess the risk of collisions between obstacles and the robotic arms, the robotic arm movement speed was smoothly adjusted.

The experiments showed that this study realized smooth robot self-collision and environment-collision avoidance, which has potential benefits for various scenarios such as medical robots and rehabilitating robots.

## 2. Materials and Methods

### 2.1. Kinematics of Coordinating Operation of Dual-Arm Robot

**Figure 1:**
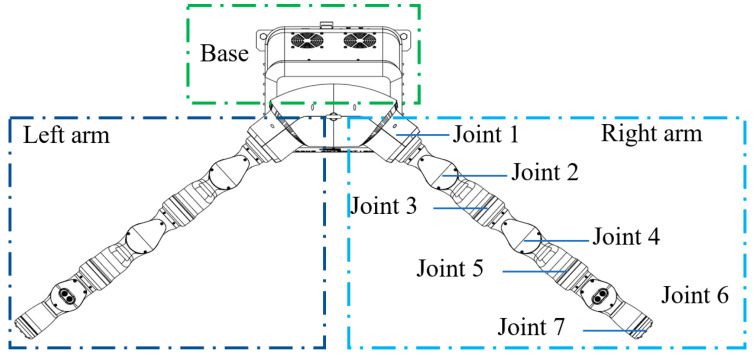
Schematic diagram of a redundant dual-arm robot.

The schematic diagram of the redundant dual-arm robot used in this study is shown in Figure 1. The dual-arm robot had a symmetric structure with seven rotating joints. Compared with the traditional 6 DoF robotic arm, the 7 DoF arm shows self-motion characteristics. When the two redundant arms coordinate to carry objects, a closed kinematic chain is formed between the left arm and the object, as well as between the object and the right arm, to constrain the movement efficiently [11,20].

**Figure 2:**
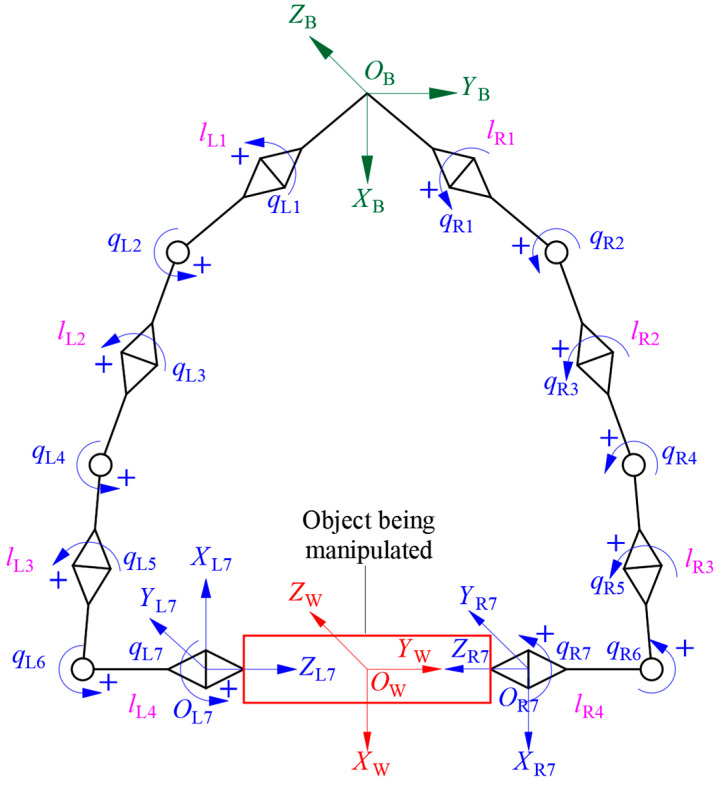
Frames of a redundant dual-arm robot.

The frames of the redundant dual-arm robot are shown in Figure 2. *O_B_*-*X_B_Y_B_Z_B_* is the base frame, and *O*_L*n*_-*X*_L*n*_*Y*_L*n*_*Z*_L*n*_ and *O*_R*n*_-*X*_R*n*_*Y*_R*n*_*Z*_R*n*_ are the frames of the end-effectors of the left and right arms, respectively. *O*_W_-*X*_W_*Y*_W_*Z*_W_ is the frame fixed at the geometric center of the object. $l_{Li}$ and $l_{Ri}$ represent the length of the *i*-th link of the left arm and that of the right arm, respectively. $q_{Li}$ and $q_{Ri}$ (*i* = 1, 2 ··· 7) are the joint angles of the *i*-th link of the left arm and those of the right arm, respectively. The symbol “+” denotes the positive direction of the rotation of a joint.

When an object is being transported, there is no relative motion between the end-effectors of the arms and the object. Therefore, the kinematic constraint equation in the coordinating operation of a redundant dual-arm robot can be derived as follows [14,20]:

(1) $$\dot{x}=J\left(q_{L},q_{R}\right)\dot{q}$$

where $\dot{x}$ is the velocity vector of the arm end-effector, $J\left(q_{L},q_{R}\right)$ is the Jacobian matrix of the coordinating operation of the dual-arm robot [21], and $\dot{q}={\left[\begin{array}{cc} {\dot{q}}_{L} & {\dot{q}}_{R} \end{array}\right]}^{T}$ is the joint angular velocity vector.

### 2.2. Shortest Distance Calculation Based on Gradient Projection Method

When the redundant dual-arm robot operates in coordination, the two arms are obstacles to each other. To avoid self-collision, it is necessary to perform calculations in real time. The shortest distance from each link of the right arm to the left arm is calculated when the right arm is regarded as the obstacle to the left arm. Furthermore, the shortest distance from each link of the left arm to the right arm is calculated when the left arm is regarded as the obstacle to the right arm. At the same time, to avoid self-collision and environment-collision, the shortest distance between the left arm, the right arm, and the environment should be calculated. A detailed derivation and a comprehensive explanation of the gradient projection method used for these distance calculations are specifically provided in Section 2.2.1, Section 2.2.2 and Section 2.2.3.

#### 2.2.1. Simplified Model of Redundant Dual-Arm Robot

Most existing robotic arm obstacle-avoidance algorithms approximate the actual arm as a segmented line and then use the gradient projection method to calculate the shortest distance between the left arm, the right arm, and the environment. However, the segmented line method mainly considers the arm length and ignores its volume element, which is often the main reason for the failure of obstacle avoidance in an actual operation. Therefore, based on the overall arm size, the actual shortest distance between the left arm, the right arm, and the environment is obtained, and the obstacle-avoidance algorithm for the redundant dual-arm robot is developed.

**Figure 3:**
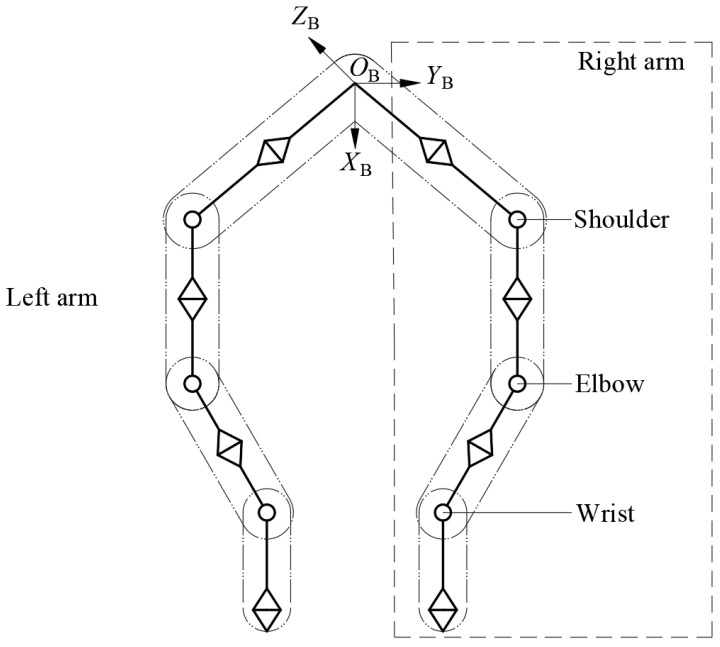
Capsule-enveloping simplified model of redundant arms of a dual-arm robot.

The common methods for simplifying the robotic arm mainly include the sphere-enveloping simplification, cuboid-enveloping simplification, capsule-enveloping simplification, and ellipsoid-enveloping simplification [22,23]. Among these, the capsule-enveloping simplification method closely approximates the actual structure of the robotic arm and provides continuous boundaries, thus improving its accuracy. Therefore, this study uses this method to simplify the dual arms, as shown in Figure 3. In the kinematic analysis, each robotic arm link is represented by a capsule consisting of a cylindrical body with hemispherical ends. The gradient projection method is used to calculate the shortest distance between capsules and between capsules and environmental obstacles. Compared to other models, the capsule model simplifies the shortest distance calculation to compute the distance between cylinders and hemispheres, avoiding high computational costs and significantly enhancing computational efficiency.

#### 2.2.2. Shortest Distance between Left Arm and Obstacle *k*

**Figure 4:**
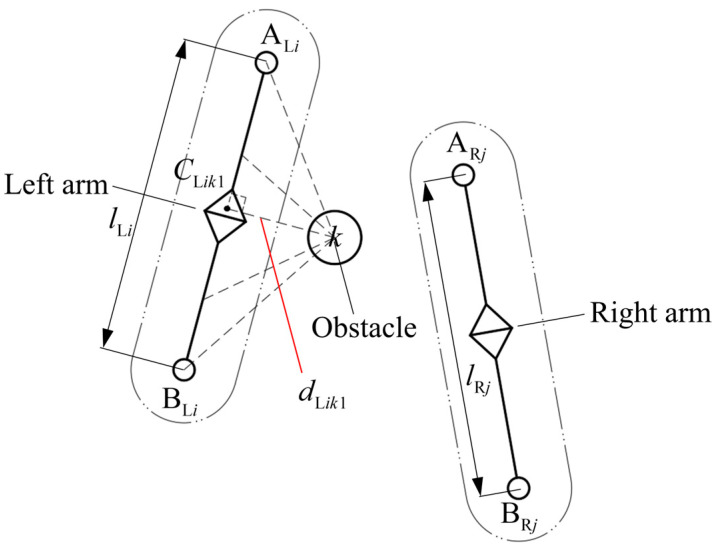
Schematic diagram of the geometric relationship between the *i*-th link of the left arm and the obstacle *k*.

To simplify the system without losing generality, the *i*-th link of the left arm, the *j*-th link of the right arm, and the surrounding obstacle *k* are taken as the objects of study to analyze the shortest distance between the *i*-th link of the left arm and the surrounding obstacle *k* when an object is being transported. The geometric relationship between the *i*-th link of the left arm and the surrounding obstacle *k* is as shown in Figure 4.

The points $A_{Li}$ and $B_{Li}$ represent the start and end joints of the *i*-th link of the left arm, and their position vectors in the base frame are ***a*** and ***b***, respectively. The length of the *i*-th link in the capsule-simplified model of the left arm is $l_{Li}$. Then, the unit vector of the *i*-th link of the left arm is

(2) $$e_{Li}=\frac{b-a}{l_{Li}}$$

The position vector of the center of the surrounding obstacle *k* located in the robot’s vision in the base frame is ***c***. The shortest point on the axis of the *i*-th link of the left arm from the obstacle *k* is recorded as $C_{Lik1}$. The position of point $C_{Lik1}$ is related to the projection value $\beta_{Lik1}$ of obstacle *k* on the axis of the *i*-th link of the left arm, and its expression is as follows:

(3) $$\beta_{Lik1}=e_{Li}^{T}\left(c-a\right)$$

where $e_{Li}^{T}$ is the transpose matrix of $e_{Li}$.

The position vector of the shortest point $C_{Lik1}$ on the axis of the *i*-th link of the left arm from the obstacle *k* in the base frame is expressed as

(4) $$g=\{\begin{array}{ccc} \;a & ; & \beta_{Lik1}<0\\ a+\beta_{Lik1}e_{Li} & ; & 0\leq \beta_{Lik1}\leq l_{Li}\\ b & ; & \beta_{Lik1}>l_{Li} \end{array}$$

Therefore, the shortest distance between the geometric center of the surrounding obstacle *k* and the axis of the *i*-th link of the left arm is given by

(5) $$d_{Lik1}=\left\|g-c\right\|$$

where ||·|| represents the modulus of the vector.

Considering the volume elements of the left arm and the surrounding obstacle, the shortest distance between the surrounding obstacle *k* and the *i*-th link of the left arm is modified as

(6) $$d_{Lik1}^{*}=d_{Lik1}-R_{Li}-R_{k}$$

where $R_{Li}$ is the radius of the *i*-th link of the left arm in the capsule-simplified model of the redundant arms, and $R_{k}$ is the radius of the surrounding obstacle *k*.

#### 2.2.3. Shortest Distance between Left Arm and Right Arm

**Figure 5:**
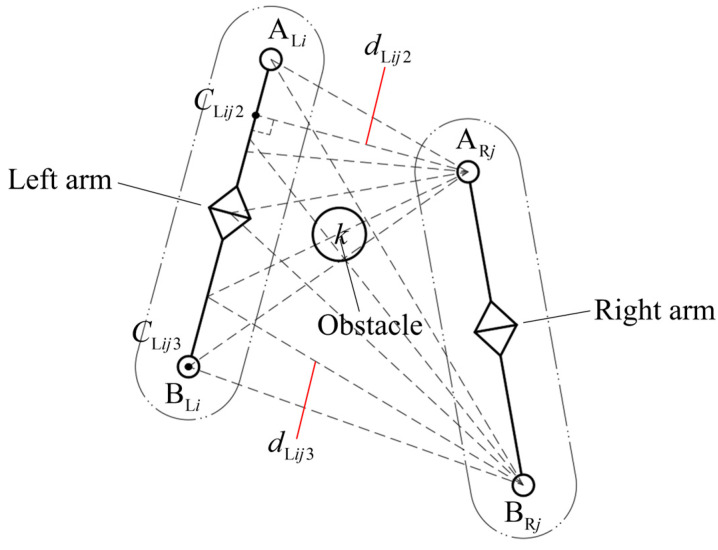
Schematic diagram of the geometric relationship between the *i*-th link of the left arm and the *j*-th link of the right arm.

When an object is being transported, the *j*-th link of the slave arm acts as an obstacle to the *i*-th link of the left arm. The shortest distance between the *i*-th link of the left arm and the *j*-th link of the right arm is analyzed, and its geometric relationship is as shown in Figure 5.

Points $A_{Rj}$ and $B_{Rj}$ represent the start and end joints of the *j*-th link of the right arm, respectively, and their position vectors in the robot’s base frame are ***h*** and ***m***. The shortest points on the axis of the *i*-th link of the left arm from the start and end joints of the *j*-th link of the right arm are recorded as $C_{Lik2}$ and $C_{Lik3}$, respectively, and their position vectors in the robot’s base frame are ***r*** and ***s***, respectively.

The start joint of the *j*-th link of the right arm is taken as the obstacle to the *i*-th link of the left arm. At this time, the projection of the start joint of the *j*-th link of the right arm on the axis of the *i*-th link of the left arm is expressed as follows:

(7) $$\beta_{Lij2}=e_{Li}^{T}\left(h-a\right)$$

According to the method in Equation (4), the shortest distance on the axis of the *i*-th link of the left arm from the start joint of the *j*-th link of the right arm is calculated as

(8) $$d_{Lij2}=\left\|r-h\right\|$$

where ***r*** is the position vector of the shortest point $C_{Lik2}$ on the axis of the *i*-th link of the left arm from the start joint of the *j*-th link of the right arm in the base frame.

Similarly, considering the volume element of the arm, the shortest distance between the *i*-th link of the left arm and the start joint of the *j*-th link of the right arm is modified as

(9) $$d_{Lij2}^{*}=d_{Lij2}-R_{Li}-R_{Rj}$$

where $R_{Sj}$ is the radius of the *j*-th link of the right arm in the capsule-simplified model of the arms.

The end joint of the *j*-th link of the right arm is taken as the obstacle to the *i*-th link of the left arm. At this time, the projection of the end joint of the *j*-th link of the right arm on the axis of the *i*-th link of the left arm is given as

(10) $$\beta_{Lij3}=e_{Li}^{T}\left(m-a\right)$$

Similarly, the shortest distance between the axis of the *i*-th link of the left arm and the end joint of the *j*-th link of the right arm can be calculated by

(11) $$d_{Lij3}=\left\|s-m\right\|$$

where ***s*** is the position vector of the shortest point $C_{Lik3}$ on the axis of the *i*-th link of the left arm from the end joint of the *j*-th link of the right arm in the base frame.

Based on the volume elements of the dual-arm robot, the shortest distance between the *i*-th link of the left arm and the *j*-th link’s end joint of the right arm is modified as

(12) $$d_{Lij3}^{*}=d_{Lij3}-R_{Li}-R_{Rj}$$

Using the above method, the shortest distance between the surrounding obstacle *k* and each link of the left arm is derived in turn, and the minimum value is recorded as $d_{L1}^{*}$. When the start and end joints of each link of the right arm are regarded as the obstacle of the left arm, respectively, the shortest distance to each link of the left arm is given, and the minimum values are recorded as $d_{L2}^{*}$ and $d_{L3}^{*}$, respectively. Therefore, the shortest distance between the left arm and all obstacles including the surrounding obstacle *k* and the link joints of the right arm is given by

(13) $$d_{0L}=\min\left\{d_{L1}^{*},d_{L2}^{*},d_{L3}^{*}\right\}$$

The point marked on the link’s axis of the left arm corresponding to the shortest distance is $p_{0L}$, called the left arm marker, and its position vector in the robot’s base frame is recorded as $p_{0L}$.

Similarly, the shortest distance between the joints of each link on the left arm and the surrounding obstacle *k* from the right arm can be provided as

(14) $$d_{0R}=\min\left\{d_{R1}^{*},d_{R2}^{*},d_{R3}^{*}\right\}$$

The point marked on the link’s axis of the right arm corresponding to the shortest distance is $p_{0R}$, called the right arm marker, and its position vector in the robot’s base frame is recorded as $p_{0R}$.

### 2.3. Obstacle-Avoidance Algorithm in Coordinating Operation

During the coordinating operation of a redundant dual-arm robot, there is inevitably a certain overlap area between the operation spaces of the left and right arms. Moreover, in a complex operating environment, the environmental obstacles inevitably affect the coordinating operation of the two arms, making self-collision and environment-collision likely and causing the coordinating operation of the arms to fail. Therefore, it is necessary to plan the trajectories and velocities of a redundant dual-arm robot. The motion-correction algorithm adjusts the redundant joints in the null space at each iteration based on the current end-effector position, target position, and obstacle information. Adjustments in the null space do not affect the end-effector trajectory, ensuring that the robot can reliably complete its tasks.

#### 2.3.1. Obstacle-Avoidance Motion Space

**Figure 6:**
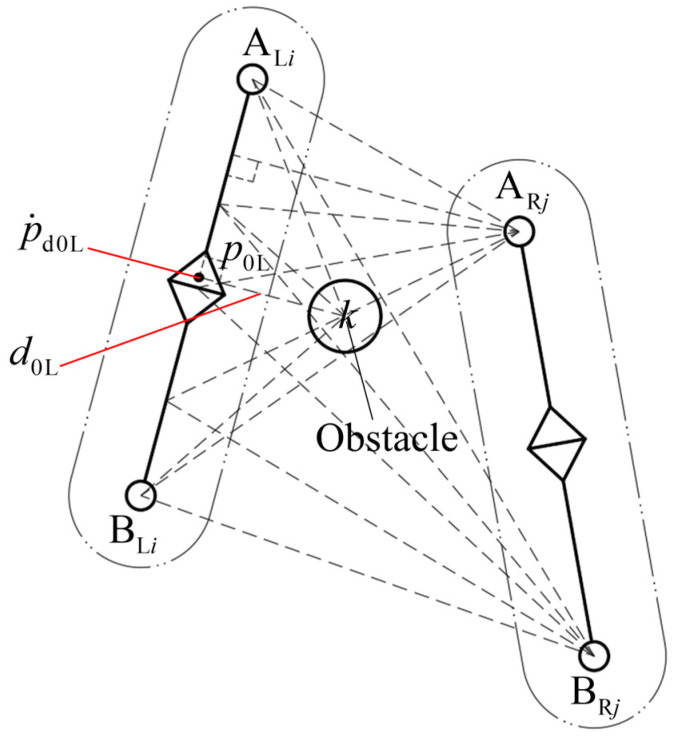
Obstacle-avoidance movement of the marker.

During the coordinating operation of a redundant dual-arm robot, if the shortest distance $d_{0L}$ between the left arm marker $p_{0L}$ and the obstacle at a specific time is less than the set “distance threshold”, the arm will generate an obstacle-avoidance action. As the arm moves in three-dimensional space, to reduce the number of computations and improve the operation efficiency of the algorithm, a reduced obstacle-avoidance motion space is introduced [13,22]. The arm only moves along the line between the geometric center of the obstacle and the marker, as shown in Figure 6. At this time, the obstacle-avoidance motion space is reduced from three dimensions to one dimension.

At this moment, the position vector of the geometric center of the obstacle with the shortest distance from the left arm to the marker $p_{0L}$ is expressed as

(15) $$d_{0L}=p_{0L}-c$$

Its unit vector is expressed as

(16) $$n_{0L}=\frac{d_{0L}}{\left\|d_{0L}\right\|}$$

The velocity ${\dot{p}}_{0L}$ and Jacobian matrix $J_{0L}$ for the obstacle avoidance of the marker on the left arm can be rewritten as

(17) $${\dot{p}}_{d0L}=n_{0L}^{T}{\dot{p}}_{0L}$$

(18) $$J_{d0L}=n_{0L}^{T}J_{0L}$$

After the reduced motion space is introduced, the marker’s velocity on the left arm to avoid obstacles in the operation space changes from ${\dot{p}}_{0L}\in R^{m\times 1}$ to ${\dot{p}}_{d0L}\in R^{1\times 1}$, and the Jacobian matrix changes from $J_{0L}\in R^{m\times n}$ to $J_{d0L}\in R^{1\times n}$.

Similarly, after the reduced motion space is introduced, the velocity and Jacobian matrix for the obstacle avoidance of the marker $p_{0R}$ on the right arm in the operation space are as follows:

(19) $${\dot{p}}_{d0R}=n_{0R}^{T}{\dot{p}}_{0R}$$

(20) $$J_{d0R}=n_{0R}^{T}J_{0R}$$

The marker’s velocity on the right arm to avoid obstacles in the operation space changes from ${\dot{p}}_{0R}\in R^{m\times 1}$ to ${\dot{p}}_{d0R}\in R^{1\times 1}$, and the Jacobian matrix changes from $J_{0R}\in R^{m\times n}$ to $J_{d0R}\in R^{1\times n}$. $n_{0R}$ is the unit vector of the position vector of the obstacle from the shortest point of the right arm to the right arm marker. The introduction of a reduced motion space dramatically reduces the number of computations.

#### 2.3.2. Obstacle-Avoidance Algorithm Theory

According to the kinematic constraint equation for the obstacle avoidance of a redundant single-arm robot in the literature [24,25], and combined with Equation (1), the obstacle-avoidance algorithm in the coordinating operation of a redundant dual-arm robot can be written as follows:

(21) $$\dot{q}=J^{+}\dot{x}+\sum_{i=L}^{R}{\left[J_{d0i}(I-J^{+}J)\right]}^{+}({\dot{p}}_{d0i}-J_{d0i}J^{+}\dot{x})$$

The superscript “+” represents the Moore–Penrose inverse of the matrix, and ***I*** is the identity matrix; $J^{+}\dot{x}$ represents the joint motion required to maintain the coordinating operation of two arms under the condition of the minimum norm solution; ${\left[J_{d0L}\left(I-J^{+}J\right)\right]}^{+}\left(p_{d0L}-J_{d0L}J^{+}\dot{x}\right)$ represents the joint motion required for the left arm to avoid obstacles under the condition of sacrificing the minimum norm solution; the matrix $J_{d0L}\left(I-J^{+}J\right)$ represents the degrees of freedom for the marker $P_{0L}$ on the left arm to avoid obstacles without generating end-effector motion. It converts the movement of the marker on the left arm to avoid obstacles from Cartesian space to the corresponding joint space motion through the pseudo-inverse solution. ${\left[J_{d0R}\left(I-J^{+}J\right)\right]}^{+}\left({\dot{p}}_{d0R}-J_{d0R}J^{+}\dot{x}\right)$ represents the joint motion required for the right arm to avoid obstacles by sacrificing the minimum norm solution, and the matrix $J_{d0R}\left(I-J^{+}J\right)$ represents the degrees of freedom for the marker $P_{0R}$ on the right arm to avoid obstacles without generating end-effector motion. It converts the movement of the marker on the right arm to avoid obstacles from Cartesian space to the corresponding joint space motion through the pseudo-inverse solution.

To ensure the effective control of the marker for obstacle-avoidance movement during the coordinating operation of a dual-arm robot, Equation (21) is modified as follows:

(22) $$\dot{q}=J^{+}\dot{x}+\sum_{i=L}^{R}\alpha_{i}{\left[J_{d0i}(I-J^{+}J)\right]}^{+}(\gamma_{i}{\dot{p}}_{d0i}-J_{d0i}J^{+}\dot{x})$$

where $\alpha_{i}\left(d_{0i}\right)$ is the velocity weight factor for the marker to avoid obstacles, $\gamma_{i}\left(d_{0i}\right)$ is the gain of the joint angular velocity, and $d_{0i}$ is the distance between the marker and the obstacle.

The distance threshold $d_{n}\left(n=1,\;2,\;3\right)$ is introduced to ensure the smooth motion of the arms in the joint frame while avoiding collisions. The relationship between the weight factors $\alpha_{i}\left(d_{0i}\right),\gamma_{i}\left(d_{0i}\right)$, and $d_{n}$ can be given by

(23) $$\alpha_{i}\left(d_{0i}\right)=\{\begin{array}{cc} 0 & d_{0i}\geq d_{3}\\ c_{0}+c_{1}d_{0i}+c_{2}d_{0i}^{2}+c_{3}d_{0i}^{3} & d_{2}\leq d_{0i}<d_{3}\\ 1 & d_{1}\leq d_{0i}<d_{2} \end{array}$$

(24) $$\gamma_{i}\left(d_{0i}\right)=\{\begin{array}{cc} 0 & d_{0i}\geq d_{3}\\ 0 & d_{2}\leq d_{0i}<d_{3}\\ k_{0}+k_{1}d_{0i}+k_{2}d_{0i}^{2} & d_{1}\leq d_{0i}<d_{2} \end{array}$$

where $d_{1}$, $d_{2}$, and $d_{3}$ are three set distance thresholds; *c*_0_, *c*_1_, *c*_2_, *c*_3_, *k*_0_, *k*_1_, and *k*_2_ are constant coefficients whose values are as follows:

$$\{\begin{array}{c} k_{0}=\frac{\gamma_{max}d_{2}^{2}}{d_{1}^{2}-2d_{1}d_{2}+d_{2}^{2}}\\ k_{1}=\frac{-2\gamma_{max}d_{2}}{d_{1}^{2}-2d_{1}d_{2}+d_{2}^{2}}\\ k_{2}=\frac{\gamma_{max}}{d_{1}^{2}-2d_{1}d_{2}+d_{2}^{2}} \end{array};\;\{\begin{array}{c} c_{0}=\frac{-d_{3}^{3}+3d_{2}d_{3}^{3}}{d_{2}^{3}-3d_{2}^{2}d_{3}+2d_{2}d_{3}^{2}-d_{3}^{3}}\\ c_{1}=\frac{-6d_{2}d_{3}}{d_{2}^{3}-3d_{2}^{2}+3d_{2}d_{3}^{2}-d_{3}^{3}}\\ c_{2}=\frac{3\left(d_{2}+d_{3}\right)}{d_{2}^{3}-3d_{2}^{2}d_{3}+3d_{2}d_{3}^{2}-d_{3}^{3}}\\ c_{3}=\frac{-2}{d_{2}^{3}-3d_{2}^{2}d_{3}+3d_{2}d_{3}^{2}-d_{3}^{3}} \end{array}$$

When $d_{0i}\geq d_{3}$, it is a safe area. Let $\alpha_{i}=\gamma_{i}=0$, meaning that the angular velocity values of the joints of the left and right arms are solved under the minimum norm solution condition. At this time, Equation (22) is rewritten as

(25) $$\dot{q}=J^{+}\dot{x}$$

When $d_{2}\leq d_{0i}\leq d_{3}$, it is an early warning area. The velocity weight factor of the marker is not introduced, meaning that $\gamma_{i}=0$. However, $\alpha_{i}$ changes according to Equation (23) so that the homogeneous solution of the joint angular velocity gradually increases from zero. At this time, the redundant arm starts to avoid collisions to ensure that the arm runs smoothly while staying away from obstacles. When $d_{0i}$ decreases to $d_{2}$, $\alpha_{i}$ reaches the maximum value $\alpha_{\max}$, and Equation (25) is rewritten as

(26) $$\dot{q}=J^{+}\dot{x}-\sum_{i=L}^{R}\alpha_{i}{\left[J_{d0i}(I-J^{+}J)\right]}^{+}J_{d0i}J^{+}\dot{x}$$

When $d_{1}\leq d_{0i}\leq d_{2}$, it is a dangerous area. With the further reduction of $d_{0i}$, the velocity gain $\gamma_{i}$ of the marker increases rapidly from zero according to Equation (24), and, thus, the homogeneous solution of the joint angular velocity changes rapidly to ensure the smooth operation of the arm while accelerating the collision-avoidance action of the arm. When $d_{0i}$ decreases to $d_{1}$, $\gamma_{i}$ reaches the maximum value $\gamma_{\max}$, and Equation (26) is rewritten as

(27) $$\dot{q}=J^{+}\dot{x}+\sum_{i=L}^{R}\alpha_{\max}{\left[J_{d0i}(I-J^{+}J)\right]}^{+}(\gamma_{i}{\dot{p}}_{d0i}-J_{d0i}J^{+}\dot{x})$$

### 2.4. Simulation Analysis

To verify the algorithm’s effectiveness, it was applied to a redundant dual-arm robot, as shown in Figure 1. The initial values of the joint angles of the redundant dual-arm robot are listed in Table 1. The velocity for avoiding obstacles at the marker on the arm was set to 15 mm/s, and the three distance thresholds were set as *d*_1_ = 15 mm, *d*_2_ = 30 mm, and *d*_3_ = 45 mm. The initial and target positions of the geometric center of the object to be handled were A = (766, 0, 556) mm and B = (441, 0, 291) mm. To ensure the safe operation of the robot, the physical movement range of each joint was restricted to prevent the robotic arm from exceeding its designed limits. Moreover, when solving the inverse kinematics of the robotic arm, the principle of minimum energy consumption was followed to select the optimal solution.

The given task requirements are as follows. The dual-arm robot was required to move the object from the initial position A to the target position B at a constant speed along a straight line by a coordinating operation and to avoid self-collisions and collisions with surrounding obstacles while keeping the posture of the object unchanged. The time *t* was set as 10 s, and two spherical obstacles with radius *r* = 50 mm were established in the workspace in the coordinating operation of the dual-arm robot. The positions of the geometric center in the base frame were O_1_ = (370, 55, −130) mm and O_2_ = (500, −110, −200) mm.

**Figure 7:**
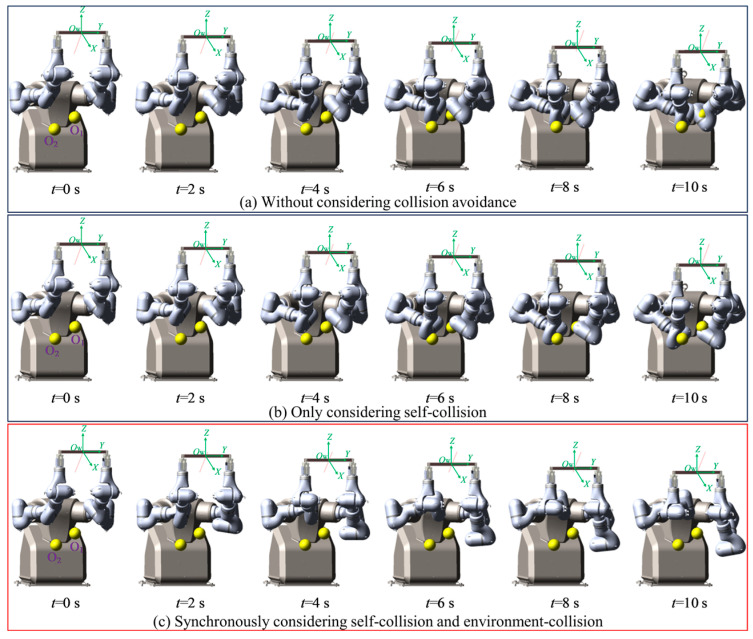
Motion trajectory in the coordinated operation of the dual-arm robot (**a**) without considering collision avoidance, (**b**) only considering the arms as obstacles to each other, and (**c**) considering the two arms as mutual obstacles and surrounding obstacles.

For the case in which the robot did not plan for obstacle avoidance, the simulation motion trajectory in the coordinated operation of the dual-arm robot is shown in Figure 7a. The two arms collided at the end of the second link during the period of *t* = 4–10 s, and the collision intensified with time. During the period of *t* = 6–10 s, the end of the second link of the master arm collided with obstacle O_1_, and the third link of the slave arm collided with obstacle O_2_. With time, the collisions between the arms and the surrounding obstacles intensified, and the coordinated operation task of the dual-arm robot failed.

When only considering the two arms as obstacles to each other, without considering the influence of surrounding obstacles on the motion of the manipulator, the simulation motion trajectory in the coordinated operation of the dual-arm robot is shown in Figure 7b. From the simulation results, it can be seen that during the period of *t* = 0–6 s, the ends of the second link of the master and slave arms gradually approached each other, and at the time where *t* = 6 s, the set safety threshold was reached. During the whole process of the coordinated operation of the dual-arm robot, there were no collisions between the links of the master arm and those of the slave arm. During the period of *t* = 6–10 s, the end of the second link of the master arm collided with obstacle O_1_, and the third link of the slave arm collided with obstacle O_2_. With time, the collisions intensified, and the coordinated operation task of the dual-arm robot failed.

It can be seen from the previous two groups of simulations that, although the end-effector of the dual-arm robot could complete the predetermined operation trajectory, collisions between each link of the master arm and those of the slave arm as well as between the links of the arms and the surrounding obstacles occurred. Therefore, in the trajectory planning in the coordinated operation of a dual-arm robot, it is necessary to realize the influence of surrounding obstacles while considering the links of the two arms to be obstacles to each other.

The trajectory planning simulation was carried out using the obstacle avoidance algorithm proposed in the process of simulation implementation. It comprehensively considered the mutual obstacles of the two arms and the surrounding obstacles. The motion simulation trajectory is shown in Figure 7c. It can be seen from the simulation results that during the entire process of the coordinated operation of the dual-arm robot, there were no collisions between the links of the two arms and no collisions between the links and surrounding obstacles. The real-time shortest distance between each link of the master arm and those of the slave arm, as well as the distances between the links of the arms and the surrounding obstacles, was always greater than the set safety threshold.

Compared with the previous three groups of experiments, it can be seen that after adopting the obstacle-avoidance algorithm proposed in this study, the redundant dual-arm robot not only completed the coordinated operation but also realized real-time self-collision avoidance and surrounding obstacle avoidance by using the self-motion characteristics of the redundant arms of the dual-arm robot. Through three groups of simulations, it was proven that the algorithm in this study is indispensable for ensuring the safety of the coordinated operation of the dual-arm robot.

## 3. Results and Discussion

### 3.1. Experimental Scenario

**Figure 8:**
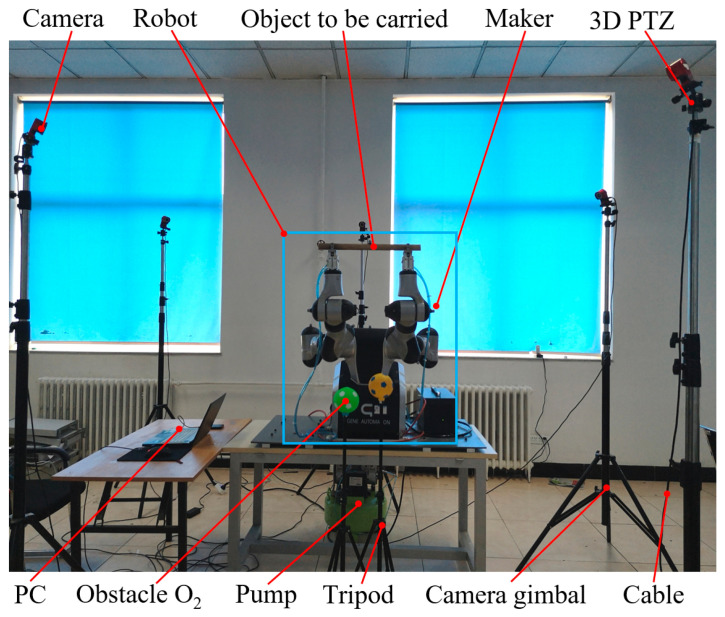
Experimental platform of coordinating operation of a redundant dual-arm robot.

To verify the obstacle-avoidance algorithm in the coordinating operation of the redundant dual-arm robot, the experimental platform shown in Figure 8 was built. The robot body was a redundant dual-arm robot from Shanghai Zhiyin Automation Technology Co., Ltd., Shanghai, China, with an operating range of 591 mm for a single arm and seven degrees of freedom for each arm. The motion capture equipment was the OptiTrack high-performance optical motion capture system produced by the American Natural Point Company, which included a camera, switch, camera gimbal, and three-dimensional (3D) PTZ (pan, tilt, zoom) system. The pneumatic grippers were installed at the ends of the dual-arm robotic arms, which gripped a cylindrical object powered by a pneumatic pump. Two small balls were placed in the workspace in the coordinating operation of the dual-arm robot to act as surrounding obstacles.

To capture the distance information between each link of the dual-arm robot and between each link and the surrounding obstacles during the 3D motion of the redundant dual-arm robot, five optical cameras of the OptiTrack motion capture device were reasonably arranged in space in this experiment, so the dual-arm robot was always within the field of view of each camera during the motion. At the same time, the markers were pasted on the outer surface of each joint of the arm and the geometric center of the object to be transported. The OptiTrack motion capture system captured the 3D motion information of these markers and fed the information to a laptop computer connected to it. The motion control frequency for the robot was set at 250 Hz, and the obstacle-avoidance algorithm was executed at 50 Hz. These values can be adjusted based on specific requirements.

### 3.2. Experimental Results

**Figure 9:**
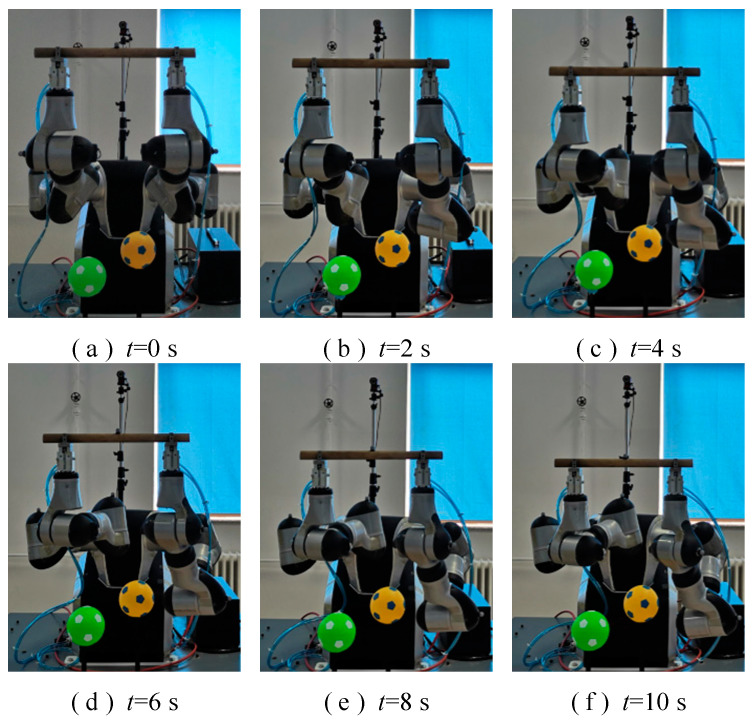
Motion trajectory for the coordinating operation of the dual-arm robot considering self-collision and environment-collision.

To make the redundant dual-arm robot run safely and stably in the process of a coordinating operation, the obstacle-avoidance algorithm proposed in this study, which comprehensively considers self-collision and environment-collision, was used to carry out a trajectory planning experiment. The motion trajectory for the coordinating operation of the dual-arm robot is shown in Figure 9.

**Figure 10:**
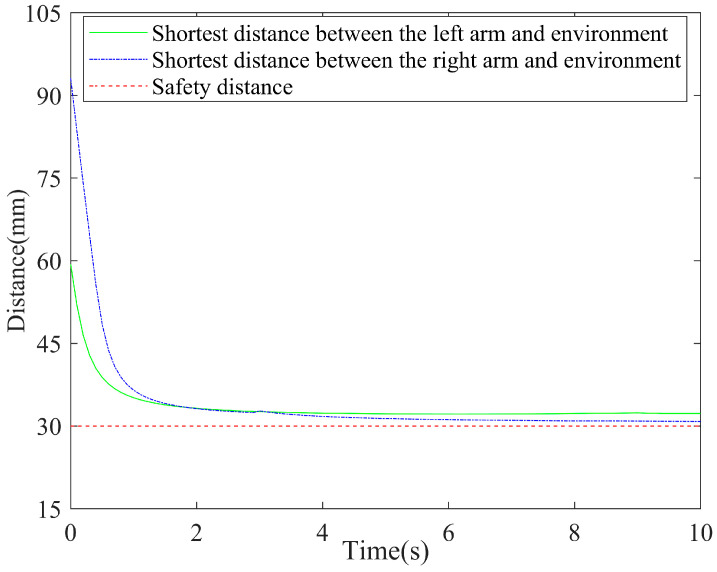
Shortest distance between the left arm, the right arm, and the environment.

**Figure 11:**
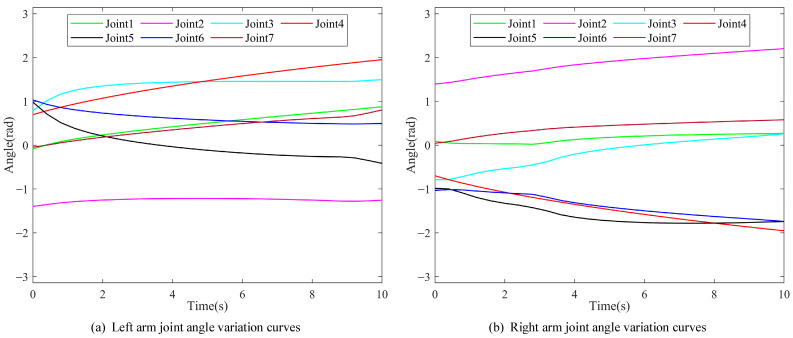
Angular variation curves for each joint of the (**a**) left arm and (**b**) right arm.

The shortest distance between the left arm, the right arm, and the environment in the entire motion process of the robot during the coordinating operation were analyzed and calculated, as shown in Figure 10. The angular changes of the joints of the left and right arms are shown in Figure 11a,b, respectively.

It can be seen from Figure 9 and Figure 10 that during the period of *t* = 0–0.2 s, the shortest distance between each link of the left arm and those of the right arm, as well as the distances between the links of the arms and the surrounding obstacles, was always greater than the threshold *d*_3_ without collision, and the dual-arm robot performed an object-holding task. The second link of the left arm approached the obstacle during the period of *t* = 0.2–3.8 s, and its shortest distance was between the thresholds *d*_3_ and *d*_2_. The system issued an early warning, and the dual-arm robot took obstacle-avoidance actions while performing the given task. At time *t* = 3.8 s, the shortest distance reached the threshold *d*_2_, and the obstacle-avoidance action was accelerated to keep away from the obstacle.

The distances between each link of the right arm and the obstacle were always greater than the threshold *d*_3_ during the period of *t* = 0.2–0.6 s, and no collision occurred. During the period of *t* = 0.4–4.6 s, the third link was close to the obstacle. When the shortest distance was between the thresholds *d*_3_ and *d*_2_, the system issued an early warning and took obstacle-avoidance actions. At time *t* = 4.6 s, the shortest distance reached the threshold *d*_2_, and the obstacle-avoidance action was accelerated to keep away from the obstacles.

Figure 10 and Figure 11a demonstrate that the second link of the left arm approached the obstacle during the period of *t* = 0.2–3.8 s, and the angle of joint 1 changed faster so that the marker on the second link moved far away from the obstacle. It can be seen from Figure 10 and Figure 11b that the third link of the right arm approaches the obstacle during the period of *t* = 0.4–4.6 s, and the angle of joint 2 changes faster so that the marker on the third link moves far away from the obstacle. It can be seen in Figure 9, Figure 10 and Figure 11 that during the entire process of the coordinating operation of the dual-arm robot, there were no self-collisions and environment-collisions. The shortest distance between the left arm, the right arm, and the environment were always greater than the set safety threshold. Additionally, the algorithm ensured smooth and continuous movements while coordinating the operation.

The effectiveness of this algorithm was validated in controlled experimental environments. However, a broad application in real-world scenarios requires addressing its limitations in unstructured environments and operational complexity. Zhao et al. [26] proposed an online policy learning method that avoids the latency of traditional offline learning by adjusting control strategies in real time, enabling quick responses to dynamic task demands. Similarly, Dong et al. [27] proposed a method for capturing and modeling complex relationships in heterogeneous networks using meta-path strategies, which excels in identifying complex patterns in data, thereby enhancing the robot’s rapid adaptation and response capabilities in complex environments. Su et al. [28] presented the robot-assisted minimally invasive surgery method, which combines teaching-by-demonstration and advanced modeling techniques. Using dynamic time warping and Gaussian mixture model-based dynamic movement primitives, it creates detailed 3D surgical skill models from multiple human demonstrations, enhancing the system’s precision in replicating complex surgical operations.

Although these methods do not directly address dual-arm robots’ obstacle avoidance, they provide significant methodological support for improving operational efficiency and safety. Future research can incorporate these methods by introducing online policy learning and meta-path-based modeling techniques into dual-arm service robots’ control systems for real-time adaptive adjustments, enhancing response speed and task execution efficiency in dynamic environments. Additionally, utilizing dynamic time warping and Gaussian mixture model techniques can improve algorithmic precision and flexibility in handling complex tasks. Integrating these advanced methods will further enhance dual-arm service robots’ obstacle-avoidance capabilities and overall performance in complex environments and operations.

## 4. Conclusions

In this study, an obstacle-avoiding algorithm for a dual-arm robot is proposed to avoid self-collision and environment-collision. The algorithm utilizes a distance threshold to assess potential collision risks between the robot’s arms and surrounding obstacles. It incorporates a velocity weight to dynamically adjust the arm’s speed, thereby ensuring smooth and continuous joint motion. Furthermore, the shortest distance calculation method proposed in this study corrects inaccuracies previously encountered during obstacle avoidance. The collision-avoidance method could provide potential benefits for various scenarios such as medical robots and rehabilitating robots.

Although this study makes a certain advancement, the algorithm was only tested in environments with static, spherical obstacles, which simplifies real-world scenarios. Future research will explore advanced control strategies to improve the safety and efficiency of dual-arm service robots in coordinated operations. The algorithm’s applicability will be extended to integrate visual detection techniques to enhance obstacle avoidance in unstructured environments. Additionally, the robot’s operational intelligence will be improved to enable more autonomous task completions in complex environments.

## Figures and Tables

**Table 1:** Initial joint angle values of a redundant dual-arm robot.

Angle	*q*_1_/(°)	*q*_2_/(°)	*q*_3_/(°)	*q*_4_/(°)	*q*_5_/(°)	*q*_6_/(°)	*q*_7_/(°)
Left	−5	−80	45	40	56.3	59.2	−2.5
Right	5	80	−45	−40	−56.3	−59.2	2.5

## Data Availability

The dataset analyzed in the current study is available from the corresponding author on reasonable request.
